# Life-saving automated external defibrillation in a teenager: a case report

**DOI:** 10.1186/1752-1947-1-76

**Published:** 2007-09-03

**Authors:** Corsino Rey, Antonio Rodríguez-Nuñez, Alberto Medina, Juan Mayordomo

**Affiliations:** 1 Paediatric Intensive Care Unit. Department of Paediatrics, Hospital Universitario Central de Asturias, University of Oviedo, Oviedo, Spain; 2 Pediatric Emergency and Critical Care Division, Department of Paediatrics, Hospital Clínico Universitario de Santiago de Compostela, Servicio Galego de Saúde (SERGAS) and University of Santiago de Compostela, Santiago de Compostela, Spain

## Abstract

**Background:**

Adolescent sudden death during sport participation is commonly due to cardiac causes. Survival is more likely when an automated external defibrillator (AED) is used soon after collapse.

**Case presentation:**

We describe a case of sudden death in a 14 year old boy with two remarkable points, successful resuscitation at school using an AED and diagnosis of arrhythmogenic right ventricular cardiomyopathy (ARVC). Bystander cardiopulmonary resuscitation (CPR) was immediately started by a witness and 5 minutes after the event the child was placed on an AED monitor that determined he was in a non shockable rhythm, therefore CPR was continued. Two minutes later, the AED monitor detected a shockable rhythm and recommended a shock, which was then administered. One minute after the shock, a palpable pulse was detected and the child began to breathe by himself. Four days after cardiac arrest, the boy was conversing and self-caring. Cardiac magnetic resonance imaging was suggestive of ARVC.

**Conclusion:**

Ventricular fibrillation secondary to ARVC may be a devastating event and places young patients and athletes at high risk of sudden death. Immediate CPR and AED have been demonstrated to be lifesaving in such events. Therefore, we suggest that schools should have teachers skilled in CPR and accessible AEDs.

## Background

Automated external defibrillators (AEDs) have been used to treat sudden cardiac arrest in the adult patient population for over 20 years. Until recently, the use of AEDs in children was not recommended. Therefore, when a paediatric patient suffered a cardiac arrest with a shockable rhythm in an out-of-hospital setting, the only available treatment was manual defibrillation that should be administered by the emergency advanced life support team on arrival, with consequent delay in treatment [1,2]. The incidence of athlete sudden deaths appears to be in the range of 1:200.000 young people of high school per year [3]. Although relatively unfrequent, such deaths are more common than previously thought and represent a substantive health problem [4]. Sudden death during sport participation is commonly due to cardiac causes. Hypertrophic cardiomyopathy, coronary artery anomalies and myocarditis are the more frequent [3]. Therefore, survival is more likely when bystander cardiopulmonary resuscitation (CPR) and AED are initiated soon after collapse. However, and despite the new international CPR guidelines that reinforce this message, few cases of successful AED in children have been reported and it seems that paediatric staff remains unaware of the potential impact of this therapy.

## Case presentation

**Figure 1:**
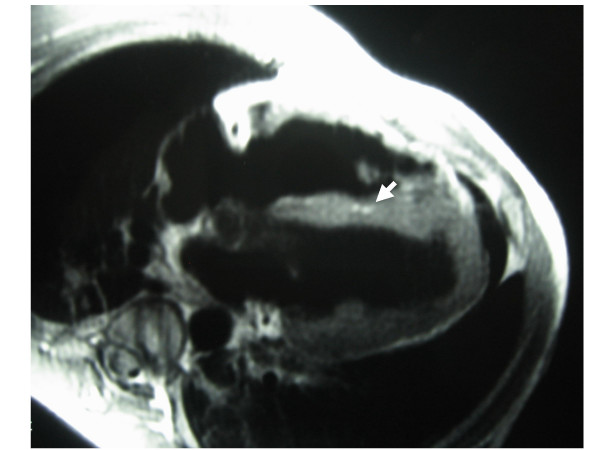
**Cardiac magnetic resonance**. Cardiac magnetic resonance showing an area of increased signal intensity compatible with myocardial fatty substitution.

**Figure 2:**
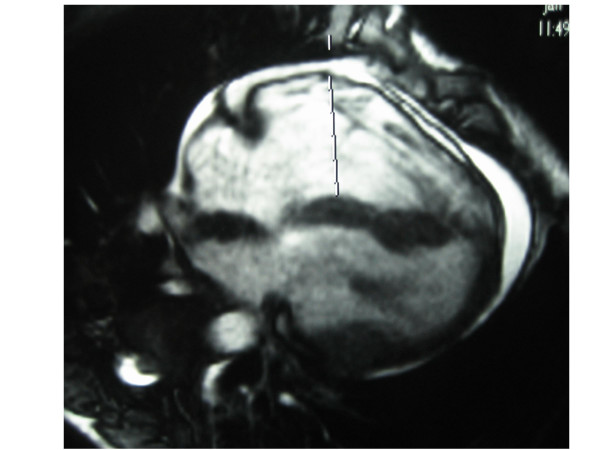
**Cardiac magnetic resonance**. Cardiac magnetic resonance showing right ventricle dilatation with increased wall thinning.

A 14-year-old boy collapsed while playing a football match at school. Bystander CPR, including both chest compressions and mouth-to-mouth resuscitation, was immediately started by a witness who was trained in basic life support (BLS), while the emergency medical system was activated by another layperson. A paramedic BLS ambulance arrived 5 minutes after the event and immediately placed the child on an AED monitor (Heartstart FR2, Philips). Initially, the child was determined to be in a non shockable rhythm, therefore CPR was continued. Two minutes later, the AED monitor detected a shockable rhythm and recommended a shock, which was then administered at 150 joules. Chest compressions and bag-mask ventilation were resumed and one minute after the shock, a palpable pulse with a rate of 85 bpm was detected and the child began to breathe by himself. Fifteen minutes after collapse, the emergency advanced life support team arrived. At that time, heart rhythm was sinus rhythm with premature ventricular beats. Because of respiratory distress, the boy was intubated and transported to a paediatric intensive care unit (PICU) for further treatment. On admission physical examination revealed normal range HR with frequent ventricular extra-systoles, normal blood pressure and adequate peripheral perfusion. Amiodarone continuous IV infusion was started. A first echocardiogram exam revealed a structurally intact heart with adequate biventricular function. Cardiac index measured by pulse contour analysis was also within normal limits. His initial laboratory evaluation revealed a serum troponin level of 0.02 (normal: 0.01 – 0.04 ng/mL) that five hours later increased to 1.17 ng/mL, returning to normal ranges 3 days after PICU admission. After cardiac evaluation, oral beta blocker therapy was started. He was on mechanical ventilation during two days and he was weaned without events. Four days after cardiac arrest, the boy was conversing and self-caring. Brain computed tomography and electroencephalogram revealed no abnormalities. Cardiac magnetic resonance imaging was suggestive of arrhythmogenic right ventricular cardiomyopathy (ARVC) (Figures 1, 2) and consequently a cardioverter-defibrillator was implanted in order to prevent a new episode of sudden death. He has subsequently returned to school with the advice to not perform vigorous exercise or engage in competitive sports.

## Discussion

We presented a case of sudden death in a 14 year old boy with two remarkable points, successful resuscitation at school using an AED and diagnosis of ARVC. Widespread introduction of AED has resulted in improved outcome from ventricular fibrillation. However, for a number of reasons, including the cost of these devices and unawareness of the importance of public access defibrillation also for children, AEDs are not found in most Spanish schools. According to current international guidelines [5], a standard AED should be used in children over 8 years of age and a device with dose attenuator should be used in children between 1 and 8 years. If no such system is available, an unmodified adult AED may be used in children older than 1 year [5,6]. In our case, a BLS trained witness started CPR until a paramedic rapid response unit with a standard AED arrived at the scene. It is well known now that time to defibrillation is the major survival factor in out-of-hospital cardiac arrest due to a shockable rhythm. Also, AEDs are easy to use by minimally trained lay responders. Therefore, we consider that AEDs should be readily accessible at schools and teachers (especially physical education coaches) should be trained to use these devices. Although the absolute risk for young athletes remains low when compared to adult population, the risk excess when compared to general population in their age group suggest the need for systems able to respond to unexpected events [7,8]. The American Academy of Pediatrics, endorsed a standardized pre-participation athletic evaluation form that presents several useful questions for cardiovascular risk assessment [9]. Also, Campbell and Berger [10] developed a standardized cardiovascular risk-assessment form, which could be used by any provider, for any child, at any age, at any time. Recently, the American Heart Association published an update of recommendations and considerations related to screening for cardiovascular abnormalities in competitive athletes [4].

Patients with ARVC usually have ventricular premature beats and non-sustained or sustained ventricular tachycardia demonstrating a left bundle branch block pattern. However, since ventricular tachycardia may also degenerate into ventricular fibrillation, sudden death may be the first manifestation of ARVC, as it was in our case. In recent years, ARVC has been more and more recognized as an important and frequent cause of ventricular tachyarrhythmias and sudden cardiac death, particularly in young patients and athletes, with apparently normal hearts [3,10]. ARVC is responsible for 3–5 % of sudden death for individuals younger than 65 years [11]. The diagnosis is based on electrocardiographic abnormalities and the identification of regional or global right ventricular dysfunction and fibrolipomatosis [12-14]. Electrocardiographic changes include inverted T waves in the right precordial leads beyond V1 in the absence of right bundle branch block. Right ventricular late potentials in the form of epsilon waves may be found on the routine 12 lead ECG [13]. An implantable cardioverter-defibrillator is indicated in selected high-risk patients with ARVC as in our case. ARVC occurs in a familial fashion in 30–50% and appears to follow autosomal dominant inheritance [11]. Therefore, it may be important to inform other family members about this fact and to instruct those members to also promote education in CPR and use of AEDs.

## Conclusion

Ventricular fibrillation secondary to ARVC may be a devastating event and places young patients and athletes at high risk of sudden death. Immediate CPR and AED have been demonstrated to be lifesaving in such events. Therefore, we suggest that schools should have teachers skilled in CPR and accessible AEDs.

## Competing interests

The author(s) declare that they have no competing interests.

## Authors' contributions

AM and JM were responsible for the diagnosis and treatment of the described patient. CR and AR-N performed the literature research and drafted the manuscript, which was read and approved by all authors in its final version.
